# Somatic complaints as a mediator in the association between body mass index and quality of life in children and adolescents

**DOI:** 10.1186/s12875-021-01562-1

**Published:** 2021-10-28

**Authors:** Hevy Hassan, Winifred D. Paulis, Patrick J. E. Bindels, Bart W. Koes, Marienke van Middelkoop

**Affiliations:** Department of General Practice, Erasmus MC University Medical Center, Room Na-1923, PO Box 2040, 3000 CA Rotterdam, The Netherlands

**Keywords:** Childhood obesity, Consultation, Obesity, Pediatrics, Primary care, Quality of life

## Abstract

**Background:**

Children and adolescents with overweight are known to have lower Quality of Life (QoL) compared to peers with a normal weight. QoL is a broad concept and is associated with many factors. A better understanding of the factors associated with QoL in children and adolescents and their impact on the association between overweight/obesity and QoL, may help to develop and improve interventions that lead to an improvement in QoL in children/adolescents with a high body mass index (BMI > 25). This study investigated the possible mediating effects of somatic complaints and general practitioner consultations in the association between overweight/obesity and QoL in children and adolescents.

**Methods:**

For the current study, cross-sectional data were used from a longitudinal study, the DOERAK cohort, collected from general practitioners’ medical files and through questionnaires**.** This cohort included 2-18 year olds with normal weight and overweight. Uni- and multivariate regression analyses were performed to gain more insight into variables associated with QoL. Mediation analyses were performed to investigate the possible mediating effects of somatic complaints and GP consultations in the association between overweight/obesity and QoL in children.

**Results:**

In the total sample of 733 participants aged 2-18 years, participants with normal weight had a significantly higher QoL (83.64, SD10.65) compared to participants with overweight (78.61, SD14.34) and obesity (76.90, SD13.63) at baseline. The multivariate analyses showed that a lower socio-economic status (SES), higher BMIz, and the presence of somatic complaints are associated with a lower QoL. The mediation analysis showed a significant effect of the indirect pathway of BMIz on QoL through somatic complaints (β = − 0.46, 95% CI[− 0.90, − 0.06]).

**Conclusion:**

BMIz has a direct impact on QoL in children and adolescents. Somatic complaints seem to mediate the effect of BMIz on QoL.

**Supplementary Information:**

The online version contains supplementary material available at 10.1186/s12875-021-01562-1.

## Background

Overweight and obesity are a major public health problem, with a high prevalence worldwide [1]. In 2018, 11.7% of the Dutch children aged 4-18 years old were overweight or obese, and 8% of 2 year olds. Obesity is more frequently seen in children (4-11 years) compared to adolescents aged 12-18 years [2]. It impacts physical health and can lead to cardiovascular, respiratory, musculoskeletal, neurological, gastrointestinal and endocrine complaints or diseases [3–5]. Furthermore, it has psychological and social consequences [3].

Children and adolescents with overweight have a lower Quality of Life (QoL) compared to peers with normal weight [6–8]. Longitudinal studies indicate that a poorer QoL seems to be a consequence of higher weight status, rather than a cause. Weight loss promoting interventions can lead to a better QoL [9]. These interventions are often multidisciplinary and also focus on other factors as improving physical activity and addressing psychosocial problems, which may also influence QoL [9]. QoL is a broad concept and certainly not only influenced by weight status, physical activity or psychosocial problems of the child or adolescent. Other factors that are important to take into account when the association between overweight and QoL is investigated, are age, socio-economic status (SES), marital status, parents’ education levels and presence of somatic complaints [10–12].

In children and adolescents in general, somatic complaints have negative impact on QoL, especially chronic pain [13, 14] and the co-occurrence with overweight or obesity enhances this effect [14]. Children and adolescents with overweight, compared to those with normal weight, are at higher risk to develop somatic complaints [3–5]. Thus, lower QoL observed in children and adolescents with overweight might be the result of a direct effect of weight status, but it is also possible that there is an indirect effect of the presence of somatic complaints in these children/adolescents [5, 15]. Somatic complaints are often measured by (self-report) questionnaires, however objective measurements will offer more reliable outcomes. In the Netherlands, when patients seek health care, the general practitioner (GP) is the first doctor to visit. Therefore, the number of GP consultations might be a good objective measurement for somatic complaints. More insight in the pathways between QoL, somatic complaints and overweight may help to develop and improve interventions that lead to an improvement in QoL in children and adolescents with overweight/obesity.

The general aim of this study was to obtain more insight in the association between overweight and obesity and QoL among children and adolescents. The specific aim of this study was to better understand factors (e.g. BMIz, somatic complaints, GP consultations) associated with QoL in children and adolescents; and their impact on the association between overweight/obesity and QoL. Secondly, the possible mediating effects of somatic complaints and GP consultations in the association between overweight/obesity and QoL in children and adolescents were investigated.

## Methods

### Study design

This study was conducted using data from the DOERAK cohort (“Determinants of (sustained) Overweight and complaints; Epidemiological Research among Adolescents and Kids in general practice”) [5, 16]. This prospective longitudinal cohort was constructed to gain more insight in the differences between children and adolescents with normal weight and overweight in Dutch general practice.

### Participant selection

The DOERAK study population was selected from 73 general practices in the region of Rotterdam, the Netherlands, between December 2010 up to April 2013. Children aged 2-18 years and their parents were asked to participate in the study when visiting their GP for any type of non-emergency complaint, during this consult they received explanation about the study. In order for children to participate, their parents required a basic knowledge of the Dutch language. Excluded were children with a mental or physical disability or comorbidities affecting their weight status. After written information was sent out, all interested children and their parents were contacted by the researchers to answer any questions and check their consent of participation. In line with Dutch law, written informed consent of parents and children > 12 years old was mandatory for participation.

### Data collection

Participants received questionnaires at baseline and during follow-up at 6, 12, and 24 months. In the absence of response, participants received a reminder after 1 week, this was repeated weekly for 8 weeks.

GPs filled out a questionnaire regarding age, sex, height, weight and waist circumference of the child, reason of consultation at baseline and of all consultations in the previous 12 months. During 2 years of follow-up, the total number and reasons of consultation were extracted from the patient files. All diagnoses were registered following the international classification of primary care, ICPC-coding [17].

### Measurements

The baseline questionnaire, based on proxy-report, informed on height and weight of the mother, SES (income higher or lower than 2000 euro/month), ethnicity (both parents born in the Netherlands, at least one parent born in another country), marital status (parents living together or separated) and education levels of the parents based on education in the Netherlands (lower (<MBO) and higher (HBO/WO)) [18].

Additionally, all questionnaires informed on QoL using the PedsQL [19] and presence of somatic complaints (Somatic Complaint List) [20]. Parents filled these out for children aged 2-9 years old and self-report was used for 10-18 year olds [21].

The PedsQL™ is a 23-item generic health-related quality of life (HRQL) questionnaire that informs on the physical (8 items), emotional (5 items), social (5 items), and school functioning (5 items) of the child. A 5-point response scale is used, which is transformed to a 0 (poorest QoL) to 100 (best QoL) scale and a total score of the mean of all items was calculated [19].

General health status was measured with the SCL, using the version validated in the Netherlands [20]. It contains 11 items and a 5-point response scale on each item. The total score (range 11-55) of the somatic complaints was comprised, with higher scores indicating more somatic complaints. The total number of GP consultations in the past 12 months (including the visit at baseline) and reason for consultation was obtained from the questionnaires filled out by GPs. The reasons for consultation were categorized into the following ICPC groups: ICPC A (General and unspecified), ICPC D (Digestive), ICPC L (Musculoskeletal), ICPC R (Respiratory), ICPC S (Skin), ICPC H (Ear), ICPC Other, ICPC Not coded.

The anthropometry was measured at baseline and during follow-up at 6, 12 months by the GP or the research assistant following the same protocols and instructions. At 24 months follow-up, self-reported height and weight in the questionnaires were used, because not all participating GPs had registered this. Height and weight was used to calculate the BMIz of the participants, using international age- and sex specific cut-offs of the BMI to classify the weight status [22].

### Statistics

Analyses were performed with SPSS (version 25.0). Descriptive statics at baseline were used to describe the study population, including the variables age, SES, parental marital status, education level of parents, BMIz, BMI of the mothers (80% of the proxy-reports at baseline were filled out by the mother of the child), somatic complaints, total consultations, and ICPC groups. For continuous variables the means (SD) were used, for the dichotomous variables the frequencies (%).

A one way ANOVA was performed to determine potential differences in QoL between the weight groups (normal weight, overweight weight and obesity).

Univariate linear regression analyses were performed to test the association between BMIz, age, SES, marital status, education level of parents, BMI of the mothers, somatic complaints, total consultations, and ICPC groups with QoL. In addition, a multivariate model was conducted which included all variables. All analyses were performed in the total population and in age subgroups (2-9 years old vs 10-18 years old).

Potential confounders for BMIz and QoL were age, SES, parental marital status, education level of parents, BMI of the mother, somatic complaints, GP consultations, and ICPC groups. Potential confounding on the association between BMIz and QoL was tested by adding one variable to the univariate model. If there was a > 10% difference in the Beta values of the BMIz, the variable was considered a confounder. The strength of the associations was expressed in Betas with 95% confidence intervals (CI).

**Fig. 1:**
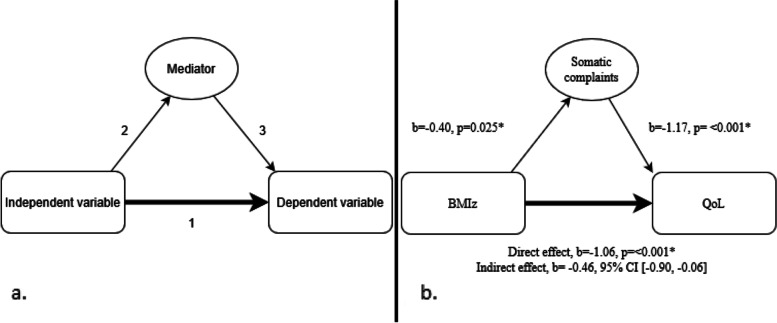
**a** Mediator analysis model for a single mediator. Natural direct effect = 1, Natural indirect effect = 2 + 3, **b** Mediation Analyses in children aged 2-18 years (DOERAK study 2010-2013). * significant p < 0.05

To test the independent relation of BMIz (independent variable, a) on QoL (dependent variable, Y), a causal mediation analysis in a model containing only one single mediator was performed (Fig. 1a). The variables “somatic complaints (mediator, m)” or “GP consultations (mediator, m)” (total number) were used as the mediating variables. A mediating variable transmits the effect of independent variables on dependent variables [23]. If there was confounding (c), it was adjusted for the confounder in the model. A causal mediation analysis was only performed if all standard criteria were met (Table 1). The direct and indirect effects were calculated by SPSS using the Andre F. Hayes function [24].

**Table 1:** Criteria for mediation analysis

Step 1:	The independent variable (a) must have a significant total effect on the dependent variable (Y).^a^
Step 2:	The independent variable must have a significant direct effect on the mediator (m).^b^
Step 3:	Use the regression coefficient from step 1 and add m as a predictor to test the effect of m on Y. Then m must have a significant direct effect on Y.^a^
Step 4:	Check whether the effect of a on Y disappears/diminishes after adding m.^a^

Use this table in combination with Fig. 1a) for a better understanding of the pathways. ^a^Pathway 1. ^b^Pathway 2

## Results

### Characteristics of the study population

Seven hundred thirty-three participants contributed to the study, 472 were aged 2-9 years old, 261 were aged 10-18 years. The mean age of the total population was 8.2 years (SD4.02), the mean BMIz was 0.12 (SD1.35) and 77.2% had a high SES. The average somatic complaints score was 19.08 (SD-5.64). Mean number of GP consultations was 4.23 (SD2.89) during the 12 months up to baseline. Compared to children, adolescents less frequently suffered from digestive (16.5% vs. 25.8%), respiratory (26.8% vs. 48.3%), or ear complaints (14.9% vs. 26.7%), but had more musculoskeletal complaints (41% vs. 13.3%) (Table 2).

**Table 2:** Baseline characteristics of study population (DOERAK study 2010-2013)

	Total study population (***N*** = 733)	Children Aged 2-9 years (***N*** = 472)	Adolescents Aged 10-18 years (***N*** = 261)
**Age (years), mean (SD)**	8.20 (4.02)	5.71 (2.25)	12.70 (2.17)
**Social-economic status (SES)**	**N (%)**	**N (%)**	**N (%)**
**Low (< 2000 euros)**	132 (22.8)	82 (21.5)	50 (19.2)
**Middle/High (≥2000 euros)**	448 (77.2)	299 (78.5)	149 (74.9)
**Education level in households**	**N (%)**	**N (%)**	**N (%)**
**High (HAVO/VWO)**	268 (42.9)	197 (48.8)	71 (32.3)
**Low (MBO)**	356 (57.1)	207 (51.2)	149 (67.7)
**Ethnicity**	**N (%)**	**N (%)**	**N (%)**
**Both parents born in Netherlands**	515 (85.4)	335 (85.9)	180 (84.5)
**At least one parent born in another country**	88 (14.6)	55 (14.1)	33 (15.5)
**Marital status**	**N (%)**	**N (%)**	**N (%)**
**Parents separated**	103 (14.1%)	52 (13.0)	51 (23.2)
**Parents together**	518 (83.4)	349 (87)	169 (76.8)
**BMI-z score, mean (SD)**	0.12 (1.35)	0.04 (1.35)	0.27 (1.33)
**BMI mothers, mean (SD)**	25.39 (4.52)	25.19 (4.44)	25.77 (4.63)
**Somatic complaints (score), mean (SD)**	19.08 (−5.64)	18.29 (4.93)	20.50 (6.50)
**Number of GP consultations (12 months + current), mean (SD)**	4.23 (2.89)	4.46 (3.04)	3.82 (2.57)
**ICPC at baseline**	**N (%)**	**N (%)**	**N (%)**
**ICPC A: General and unspecified**	126 (17.2)	92 (19.5)	34 (13.0)
**ICPC D: Digestive**	165 (22.5)	122 (25.8)	43 (16.5)
**ICPC H: Ear**	165 (22.5)	126 (26.7)	39 (14.9)
**ICPC L: Musculoskeletal**	170 (23.2)	63 (13.3)	107 (41.0)
**ICPC R: Respiratory**	298 (40.7)	228 (48.3)	70 (26.8)
**ICPC S: Skin**	353 (48.2)	236 (50.0)	117 (44.8)
**ICPC Other**	278 (37.9)	164 (34.7)	114 (43.7)
**ICPC Not coded**	260 (37.2)	176 (38.9)	84 (34.1)

Missing values of the total study population social economic status (SES) N = 153; Education level in households N = 109; Ethnicity N = 130; Marital status N = 112; BMIz N = 18; BMI mothers N = 115; Somatic complaints N = 149; Total consultations N = 8; ICPC Not coded at baseline N = 35

### Quality of life at baseline and during follow-up

**Fig. 2:**
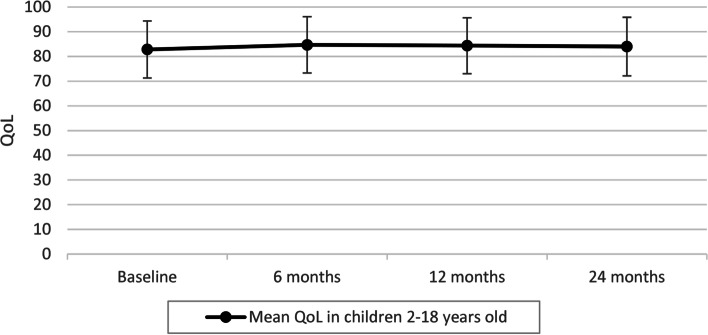
Quality of life of children during 24 months of follow-up (DOERAK study 2010-2013)

**Fig. 3:**
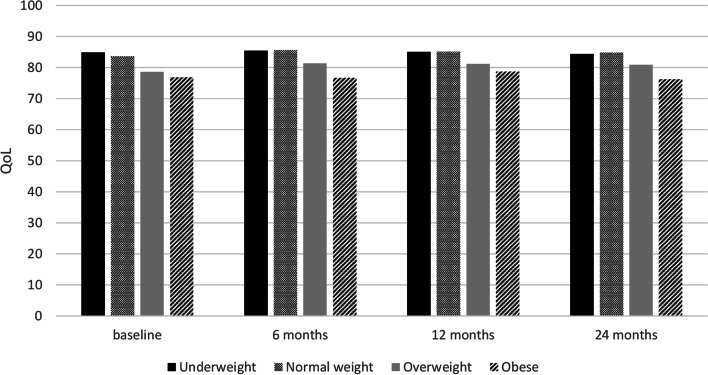
Quality of life at baseline and during follow-up 6, 12 and 24 months in children with different weight status. A higher score means a better Quality of life (DOERAK study 2010-2013)

The mean QoL scores of all participants during 2 year follow-up are presented in Fig. 2. Figure 2 presents the differences between the weight categories in QoL at the different time points. Children and adolescents with normal weight had a higher QoL (83.64, SD10.65) compared to those with overweight (78.61, SD14.34) and obesity (76.90, SD13.63) at baseline (Fig. 3). There were statistically significant differences between group means as determined by one-way ANOVA (F(3.59) = 8.36, *p* = < 0.01)”). A Tukey post hoc test showed that the group with normal weight had a significantly higher QoL compared to the group with overweight and obesity (*p* < 0.01vs. *p* = 0.02). No significant differences were seen between the group with overweight vs. obesity (*p* = 0.91). Similar differences were seen during follow-up.

### Factors associated with quality of life

The univariate analyses showed a significant association between QoL and BMIz, age, SES, household education level, marital status, somatic complaints and BMI of mothers (Table 3). The multivariate analysis showed that a higher BMIz (β = − 1.02, 95%CI[− 1.66, − 0.37]) and a higher score on the somatic complaints list (β = − 1.21, 95%CI[− 1.38, − 1.05]) were associated with a lower QoL. Higher age was associated with higher QoL (β = 0.38, 95%CI[0.12, 0.64]). Low SES was associated with a lower QoL (β = − 2.88, 95%CI[− 5.37, − 0.40]). The analyses in the different age groups are presented in supplementary documents 1 and 2. SES, marital status of parents, and parental education were associated with QoL at younger age, but no longer in the older age group.

**Table 3:** Variables associated with Quality of Life in children aged 2-18 years old (DOERAK study 2010-2013)

	Univariate			Multivariate		
	B	95% CI	***P*** value	B	95% CI	***P*** value
**Age (years)**	−0.27	−0.50, − 0.47	0.02*	0.38	0.12, 0.64	0.004*
**SES, (Low (< 2000 euros))**	−4.42	−6.71, −2.14	< 0.001*	−2.88	−5.37, − 0.40	0.02*
**Education (Low (MBO))**	−3.38	−5.22, −1.54	< 0.001*	−1.65	− 3.47, 0.17	0.08
**Ethnicity (At least one parent born in another country)**	−0.70	−3.35, 1.95	0.60	1.24	−1.20, 3.69	0.32
**Marital status (Parents separated)**	−2.94	−5.42, −0.46	0.02*	0.34	−2.24, 2.91	0.80
**BMIz score at baseline**	−1.5	−2.14, −0.77	< 0.001*	− 1.02	− 1.66, − 0.37	0.002*
**BMI mothers at baseline**	− 0.24	−0.45, − 0.04	0.02*	−0.02	− 0.22, 0.18	0.83
**Somatic complaints**	− 1.19	− 1.33, − 1.05	< 0.001*	−1.21	− 1.38, − 1.05	< 0.001*
**Number of GP consultations**	− 0.17	− 0.48, 0.15	0.29	0.22	−0.21, 0.64	0.32
**ICPC A: General and unspecified**	0.07	−2.37, 2.52	0.95	0.64	−1.69, 2.97	0.59
**ICPC D: Digestive**	−0.26	−2.43, 1.91	0.81	1.64	−0.52, 3.80	0.14
**ICPC L: Musculoskeletal**	−1.52	−3.74, 0.71	0.18	−1.17	−3.44, 1.11	0.31
**ICPC R: Respiratory**	0.75	−1.10, 2.59	0.43	0.67	−1.29, 2.63	0.50
**ICPC S: Skin**	0.85	−0.97, 2.67	0.36	−0.09	−1.95, 1.78	0.93
**ICPC H: Ear**	−1.72	−3.90, 0.46	0.12	−1.70	−3.93, 0.52	0.13
**ICPC Other**	−1.83	−3.71, 0.05	0.06	−0.87	−2.75, 1.00	0.36
**ICPC Not coded**	−2.57	−4.46, −0.67	0.01*	−1.43	−3.45, 0.59	0.17

* *P* < 0.05

### Mediators

A significant association was found in the direct (BMIz➔QoL) and indirect (BMIz➔Somatic complaints➔QoL) pathway, therefore a mediation analysis was performed (Fig. 1b).

BMIz was significantly associated with somatic complaints (β = − 0.40, 95%CI[0.05, 0.74]). BMIz and somatic complaints were significantly associated with QoL (β = − 1.06, 95%CI[− 1.63, − 0.48] vs. β = − 1.17, 95%CI[− 1.30, − 1.03]). The explained variance of the regression was statistically significant (R^2^ = 0.35, F(87.17) = 152.25, *p* < 0.001).

The indirect pathway in this analysis showed a significant effect of BMIz on QoL through somatic complaints (β = − 0.46, 95%CI[− 0.90, − 0.06]).

An association between BMIz and GP consultations was seen (β = 0.24, 95%CI[− 0.06, 0.42], however no mediation analysis was performed for the number of GP consultations as the association between GP consultations and QoL was not significant (β = − 0.10, 95%CI[− 0.41, 0.22]).

When these associations were tested in different age subgroups (2-9 years old vs. 10-18 years old), the criteria for a mediation analysis were not met.

## Discussion

The present study shows that a lower SES, higher BMIz, and the presence of somatic complaints are associated with a lower QoL. In-depth analysis indicates that the presence of somatic complaints mediates the association between BMIz and QoL in children and adolescents. However, another possible measure to indicate general health, i.e. the number of GP consultations, does not mediate this association.

Previous literature shows that many factors are linked to QoL in children and adolescents [6], including SES, BMIz and somatic complaints. This seems to be in line with our findings, since we found a strong association between BMIz and QoL [8, 9]. Different variables can in turn be associated with BMIz [25]. Somatic complaints are more frequently seen in children with a relatively high BMIz [3–5, 26]. It has been shown that the presence of somatic complaints may negatively impact weight loss interventions and thus lead to less reduction in BMIz [27]. Moreover, it is known that somatic complaints are associated with QoL [13, 14]; i.e., BMIz has a direct impact on QoL, but QoL may also be influenced indirectly through the mediating effect that somatic complaints have on the BMIz. We applied a mediation analysis to capture the complex reality with a simplified model in order to better understand the directions and associations related to QoL in children and adolescents. Mediation analysis showed an indirect pathway of BMIz on QoL for somatic complaints. This indicates that although there is a direct association between a high BMIz and lower QoL, this association is mediated by an increase in somatic complaints, that indirectly affects QoL in a negative way. Therefore, the impact of somatic complaints on QoL of children and adolescents, and especially those with overweight/obesity should always be regarded when investigating QoL in children and adolescents.

Previous research shows that higher QoL is associated with better general health status [26]. As a reflection of general health, two variables were used in the present study: somatic complaints and the number of GP consultations in the last 12 months. While somatic complaints are self-reported in a questionnaire, the number of GP consultations is an objective measure. It is known that children and adolescents with overweight/obesity visit their GP more often compared to peers with normal weight [28]. Current results show an association between BMIz and GP consultations. Though, contrary to our assumptions, no association between GP consultations and QoL was found and therefore no mediation analysis was performed. A possible explanation for this could involve that the questionnaires used to investigate somatic complaints reflect complaints that occur on daily basis. Many of the reported complaints may however not have been serious enough to lead to consulting a GP, but do appear to impact QoL. The reason for GP consultations may be of more transient nature, though more intense, and might therefore have less impact on overall QoL. Number of GP consultations therefore do not seem a suitable objective representation for the presence of somatic complaints.

The reason for consultation, instead of the number of GP consultations, might be a better measurement for the presence of somatic complaints. However, no association between reason for GP consultation and QoL was found in this study, using the before mentioned ICPC categories. It is known that presence of (chronic) pain is strongly associated with QoL [13, 14], but this measure is not represented in the ICPC categories used in the present study. It might therefore be considered to focus future research on multiple consultations for the same type of complaint, as a measure of chronic pain.

Given the wide age range of our study sample, the analyses in subgroups of children aged 2-9 years and adolescents aged 10-18 years were repeated and some differences were found. Variables SES, parental marital status, and parental education were no longer significantly associated with QoL in the adolescents. These differences could be explained by having used self and proxy-report questionnaires in adolescents versus proxy-reports in children [29]. Moreover, no mediation analyses could be performed for both subgroups, since the association between BMIz and somatic complaints was no longer significant. However, the direction and magnitude of the effects were comparable to those found in the total study population and it can therefore be argued that the differences found in the subgroup analyses are likely due to a lack of power.

### Strengths and limitations

Strengths of this study include the study population of 733 participants, with carefully collected prospective data and 2 years follow-up from a large region in the Netherlands from multiple GP practices. Anthropometry was done by a GP or research assistant and medical files could be accessed for retrieving data.

The study sample included a wide age range. However, when repeating the mediation analyses in the separate age subgroups, the direction and magnitude of the effects were comparable to those found in the total study population. As the QoL data remained stable during the 2-year follow-up of this study (Fig. 2) and there were relatively more missing data during follow-up, a limitation is that only cross-sectional data from baseline were used for the current analyses. Using follow-up data, however, would not provide additional information in order to answer our research question.

Some selection bias may have occurred, compared to the Dutch population a higher education level of household and more often both parents born in the Netherlands was found [30]. Higher education levels may have led to a higher mean QoL in this cohort compared to the overall population, as according to our analyses and past literature, education level seem to be associated with QoL [6].

This study has a smaller sample size than aimed for [5], therefore a power problem might have been the reason that some findings only showed a trend towards a significant association in the mediation analyses.

## Conclusion

In conclusion, BMIz has a direct impact on QoL in children and adolescents. Somatic complaints seem to mediate the effect of BMIz on QoL. More research on the impact of somatic complaints and BMIz, and their association with QoL is necessary. Understanding the pathways may help to develop and improve interventions that lead to an improvement in QoL of children and adolescents with overweight/obese. It is important that further research into the factors associated with QoL should incorporate a larger sample size. To provide more insight into the long-term changes in overweight/obesity and QoL in children and adolescents a longer follow-up period should be considered.

## Supplementary Information

**Additional file 1: Supplementary 1** Associations with Quality of Life in children aged 2-9 years old (DOERAK study 2010-2013). **Supplementary 2** Associations with Quality of Life in adolescents aged 10-18 years old (DOERAK study 2010-2013).

## Data Availability

The datasets used and/or analysed during the current study are available from the corresponding author on reasonable request.

## Abbreviations

QoL: Quality of Life
BMI: Body Mass Index
BMIz: Body Mass Index z-score
GP: General practitioner
SES: Socio-economic status
ICPC-coding: International classification of primary care
SCL: Somatic Complaint List
HRQL: Health-related quality of life
MBO: Middelbaar beroepsonderwijs
HBO: Hoger beroepsonderwijs
WO: Wetenschappelijk
