# Supplementary material for: Somatic complaints as a mediator in the association between body mass index and quality of life in children and adolescents
Source: BMC Fam Pract. 2021 Oct 28;22:214. doi: 10.1186/s12875-021-01562-1 (PMC8555287; doi:10.1186/s12875-021-01562-1)
Supplement: Supplementary file 1 — Additional file 1: Supplementary 1 Associations with Quality of Life in children aged 2-9 years old (DOERAK study 2010-2013). Supplementary 2 Associations with Quality of Life in adolescents aged 10-18 years old (DOERAK study 2010-2013). [file 12875_2021_1562_MOESM1_ESM.pdf]

Somatic complaints as a mediator in the association between body mass index and Quality of Life in children and adolescents.

Hevy Hassan- MSc1, Winifred D Paulis- PhD.1, Patrick JE Bindels- Prof. PhD1. , Bart W Koes- Prof. PhD.1, Marienke van Middelkoop- PhD1.

Author affiliation

1. Department of General Practice, Erasmus MC University Medical Center, PO Box 2040, 3000 CA,, Rotterdam, The Netherlands.

Correspondence to Mrs. H Hassan.

Department of General Practice, Erasmus MC University Medical Center, Room Na-1923, PO Box 2040, 3000 CA,, Rotterdam, The Netherlands. E-mail: h.hassan@erasmusmc.nl

Supplementary 1 – Associations with Quality of Life in children aged 2-9 years old (DOERAK study 2010-2013)						
	Univariate			Multivariate		
	B	95% CI	P value	B	95% CI	P value
**Age (years)**	-0.89	1.36, -0.42	<0.001*	-0.53	-1.04, -0.03	0.04*
**SES, (Low (<2000 euros))**	-5.61	-8.27, -2.94	<0.001*	-4.43	-7.48, -1.38	0.005*
**Education (Low (MBO))**	-3.64	-5.76, -1.51	0.001*	-2.04	-4.21, 0.13	0.07
**Ethnicity (At least one parent born in another country)**	-2.13	-5.28, 1.01	0.18	-2.04	-4.21, 0.13	0.71
**Marital status (Parents separated)**	-3.78	-7.00, -0.56	0.02*	0.86	-2.58, 4.29	0.63
**BMIz score at baseline**	-0.91	-1.73, -0.09	0.03*	-0.64	-1.42, 0.14	0.11
**BMI mothers at baseline**	-0.14	-0.39, 0.10	0.25	0.04	-0.20, 0.28	0.73
**Somatic complaints**	-1.19	-1.38, 1.01	<0.001*	-1.04	-1.26, -0.83	<0.001*
**Number of GP consultations**	-0.02	-0.38, 0.33	0.90	0.13	-0.36, 0.62	0.59
**ICPC A: General and unspecified**	1.14	-1.61, 3.88	0.42	0.78	-1.86, 3.42	0.56
**ICPC D: Digestive**	0.95	-1.49, 3.39	0.44	2.96	0.46, 5.47	0.02*
**ICPC L: Musculoskeletal**	-3.88	-7.13, -0.63	0.02*	-2.51	-5.74, 0.72	0.13
**ICPC R: Respiratory**	0.25	-1.90, 2.40	0.82	-0.74	-3.08, 1.59	0.53
**ICPC S: Skin**	0.94	-1.20, 3.09	0.39	-0.29	-2.54, 1.96	0.80
**ICPC H: Ear**	-2.63	-5.04, -0.22	0.03*	-1.58	-4.18, 1.01	0.23
**ICPC Other**	0.01	-2.25, 2.27	0.99	-1.14	-3.41, 1.13	0.33
**ICPC Not coded**	-3.00	-5.21, -0.79	0.08*	-1.44	-3.88, 1.00	0.25

*P<0.05

Supplementary 2 – Associations with Quality of Life in adolescents aged 10-18 years old (DOERAK study 2010-2013)

	Univariate			Multivariate		
	B	95% CI	P value	B	95% CI	P value
**Age (years)**	0.24	-0.54, 1.02	0.55	1.65	0.95, 2.36	<0.001*
**SES, (Low (<2000 euros))**	-2.18	-6.48, 2.12	0.32	-0.77	-4.93, -3.39	0.72
**Education (Low (MBO))**	-2.58	-6.27, 1.10	0.17	0.30	-2.98, 3.60	0.86
**Ethnicity (At least one parent born in another country)**	1.87	-2.96, 6.70	0.45	1.73	-2.47, 5.93	0.42
**Marital status (Parents separated)**	-1.66	-5.74, 2.42	0.42	-0.48	-4.30, 3.35	0.81
**BMIz score at baseline**	-2.38	-3.61, -1.16	<0.001*	-1.81	-2.93, -0.92	0.002*
**BMI mothers at baseline**	-0.40	-0.76, -0.30	0.03*	-0.06	-0.42, 0.30	0.74
**Somatic complaints**	-1.23	-1.44, -1.03	<0.001*	-1.41	-1.67, -1.15	<0.001*
**Number of GP consultations**	-0.63	-1.27, 0.02	0.06	0.42	-0.47, 1.31	0.35
**ICPC A: General and unspecified**	-3.11	-8.16, 1.95	0.23	-2.56	-7.28, 2.15	0.29
**ICPC D: Digestive**	-4.05	-8.57, 0.47	0.08	-1.33	-5.51, 2.85	0.53
**ICPC L: Musculoskeletal**	1.05	-2.41, 4.51	0.55	-0.42	-3.92, 0,69	0.81
**ICPC R: Respiratory**	1.15	-2.63, 4.93	0.55	2.99	-0.61, 6.59	0.10
**ICPC S: Skin**	0.61	-2.75, 3.97	0.71	-0.49	-3.69, 2.72	0.77
**ICPC H: Ear**	0.04	-4.74, 4.83	0.99	-3.62	-7.92, 0.69	0.10
**ICPC Other**	-4.82	-8.16, -1.47	0.01*	-0.94	-4.33, 2.44	0.58
**ICPC Not coded**	-1.92	-5.52, 1.68	0.29	-1.61	-5.18, 1.97	0.38

*p<0.05
